# Access to Assets and Influence: Governance and Livelihoods in Protected Areas of the Annapurna and Everest Regions, Nepal

**DOI:** 10.1007/s10745-025-00643-4

**Published:** 2025-09-19

**Authors:** Jonathan H. Hanson

**Affiliations:** 1 School of Social Sciences, Education and Social Work, College Green, Queen’s University Belfast, Belfast, UK; 2 Department of Geography, Downing Place, University of Cambridge, Cambridge, UK; 3 Snow Leopard Conservancy, San Francisco, USA

## Abstract

Calls to expand the global Protected Area (PA) network to halt biodiversity loss have considerable social implications, especially for rural communities resident in or around them. Particularly in the Global South, where power imbalances between communities and conservation authorities can be more acute, PA governance approaches and limits on livelihood activities vary. Employing a theoretical framework integrating political ecology, access theory, and the Sustainable Livelihoods model, I profiled the livelihoods of households in Nepal’s Annapurna Conservation Area (ACA), with a more decentralized governance model, and Sagarmatha National Park (SNP), with a more centralized management approach. In 2014, 705 household questionnaires were collected, alongside 70 interviews for cross-methods triangulation. Livelihood scores were significantly higher in ACA, to which the park’s co-management approach has contributed through the creation of social capital, or influence. Linear regression models found that larger households and access to income from tourism best explained higher household livelihood scores, with revenue from livestock making an essential contribution in ACA but not SNP. Despite significant constitutional changes in Nepal since the data were collected, the relationship between PA governance and local livelihoods remains a critical issue for conservation in the country, the region, and the world. Access to influence, via involvement in PA governance, can improve access to multiple asset classes and therefore contribute to more positive socio-economic outcomes. In the context of achieving protected area targets in a socially-just manner, and in generating and maintaining social support for their governance, including and empowering local communities is therefore essential.

**Supplementary Information:**

The online version contains supplementary material available at 10.1007/s10745-025-00643-4.

## Introduction

### Conservation Governance

The governance of conservation involves numerous competing themes and trends. Recent proposals to protect up to 50% of the world’s surface area for biodiversity (Dinerstein et al., 2017), which may affect up one billion people, have generated controversy for their potential impacts on rural communities and livelihoods (Schleicher et al., 2019). Implicit, and often explicit, in this approach to conservation governance has been a longstanding obsession with ‘wilderness’ and ‘pristine’ nature. This has shaped Protected Areas (PA) and their governance throughout their history, often through the exclusion of traditional users (Oldekop et al., 2016). Furthermore, these users – whether peasant farmers or pastoralists – have often been deemed to be both socio-economically backward and ecologically destructive (Kreutzmann, 2012; Sandhu & Sandhu, 2015). This is often despite evidence to the contrary that many such communities have persistently and sustainably used, transformed, and resided amongst nature at specific sites for long periods of time (Tyrrell et al., 2024).

PAs are one of the most widely implemented forms of conservation governance (Elleason et al., 2021). PAs have often been gazetted and governed with a centralized or ‘top-down’ approach, so called ‘fences and fines’ or ‘fortress’ conservation (West et al., 2006). This has been the case particularly where local opposition has been weak (Adams & Hutton, 2007). However, this approach has been criticized for its lack of attention to human rights and livelihoods (Elleason et al., 2021; Tyrrell et al., 2024), such as community relocations with insufficient compensation (Pandey et al., 2024) or impacts on livelihoods (Velho et al., 2019). More recently, its effectiveness in achieving conservation goals has also been questioned (Elleason et al., 2021), while, in their meta-analysis of 165 PAs, Oldekop et al. (2016) noted a positive relationship between socio-economic and conservation outcomes.

Developing in parallel to a more exclusionary approach has been community-based conservation (CBC) or community-based natural resource management (CBNRM) in and around PAs (Baral et al., 2007). These decentralized approaches – often termed ‘bottom-up’ conservation or co-management – extend the governance of biodiversity to include local people and their institutions, with varying degrees of involvement and influence (Elleason et al., 2021). Both bottom-up and top-down governance approaches can elicit mixed responses from people who live in and around PAs (Karanth & Nepal, 2012). For instance, the poor are often particularly dependent on natural resources (Angelsen et al., 2014) and the creation and management of PAs, especially under centralized conditions, can impose critical restraints on their livelihood needs (Velho et al., 2019). But regardless of the management model, a key observation from the debate on PA governance is the importance of developing participation, social capital, and effective institutions to include rather than exclude local communities (Masud & Khan, 2024; Pandey et al., 2017; Thorn et al., 2020).

In Nepal, the period from 1958 to 1991 was marked by an exclusionary approach to PA gazettement and governance, with some displacement of resident communities (Pandey et al., 2024). In contrast, the 1990s was marked by a progressive shift toward a community-based approach to conservation, with the devolution of some authority and income generation toward local institutions (Mehta & Heinen, 2001). This included the designation of buffer zones around most National Parks in the mid-1990s, following amendments to the 1973 National Park and Wildlife Conservation Act (Budhathoki, 2004), such as at Sagarmatha (Everest) National Park (SNP) (Silwal et al., 2022). It also included the growth of community forestry (Nepal et al., 2020) and community-based tourism (Allendorf & Gurung, 2016; Hanson et al., 2023), often in and through the designation of Conservation Area, rather than National Park, PAs, such as Annapurna Conservation Area (ACA) (Dangi et al., 2018; Nepal et al., 2022). However, Howlader and Ando (2020) analyzed the effects of new PAs created in Nepal between 1995 and 2003 and noted that they reduced wood consumption by 20–40%, with stricter governance having a greater impact on this trend.

Following the implementation of such policies, many positive conservation and development outcomes, particularly the growth of grassroots organizations, were noted (Parker & Thapa, 2012). In certain cases, these also included more positive attitudes towards PA authorities (S. Nepal & Spiteri, 2011), though the links between conservation and development benefits were not always clear (Nepal et al., 2022). In the aftermath of the civil war, conservation in Republican Nepal continued its pivot toward CBC, thereby maintaining the trajectory begun in the 1990s, but introducing the concept of land zoning (Nepal et al., 2020). Aryal et al. (2021) categorize the post-2000 era of conservation in Nepal as ‘Landscape Level Participatory Management’.

More recently, however, constitutional changes in the country 2015–2017 have sought to devolve power to local levels under a more federalized system (Chaudhary, 2019). This enactment of enhanced local governance policies aimed to redefine power dynamics, access to assets, and decision-making authority at the local level. The implications of this for PAs and their governance are still unfolding. However, the risk that much of the established participatory, community-based governance of PAs and natural resources be “pulled up” to, and therefore undermined by, the newly established local or provincial governance structures has been highlighted (Thakali et al., 2018).

### Theoretical Framework

Emanating from this historical context, more authoritarian aspects of contemporary conservation have been negatively critiqued by political ecology (Brockington et al., 2006). This has been assisted by broadening of the scope of conservation, away from a limited focus on localized threats to ‘pristine’ landscapes (Robbins, 2019), to include a more globalized synthesis of drivers of biodiversity loss and its links to political economy (Adams & Hutton, 2007; Berger et al., 2013; Kelman, 2013).

Although distinct from political ecology, access theory has a similar critical function. By broadening the range of social relations that contribute to or constrain benefits from resources beyond property relations, as well as beyond rigid Marxist notions of class, access theory allows processes and relationships of access to be analyzed (J. C. Ribot & Peluso, 2003). Crucially, it also considers how access to influence, and the shaping of the political-economic context that may result, is a key component of the process and theory (Ribot, 2014). By its focus on power, access theory links clearly to political ecology. As direct access to natural capital becomes less important, the importance of access to other assets to maintain livelihood strategies by individuals, households and communities can increase (Angelsen et al., 2014; Bebbington & Perreault, 1999).

**Fig. 1:**
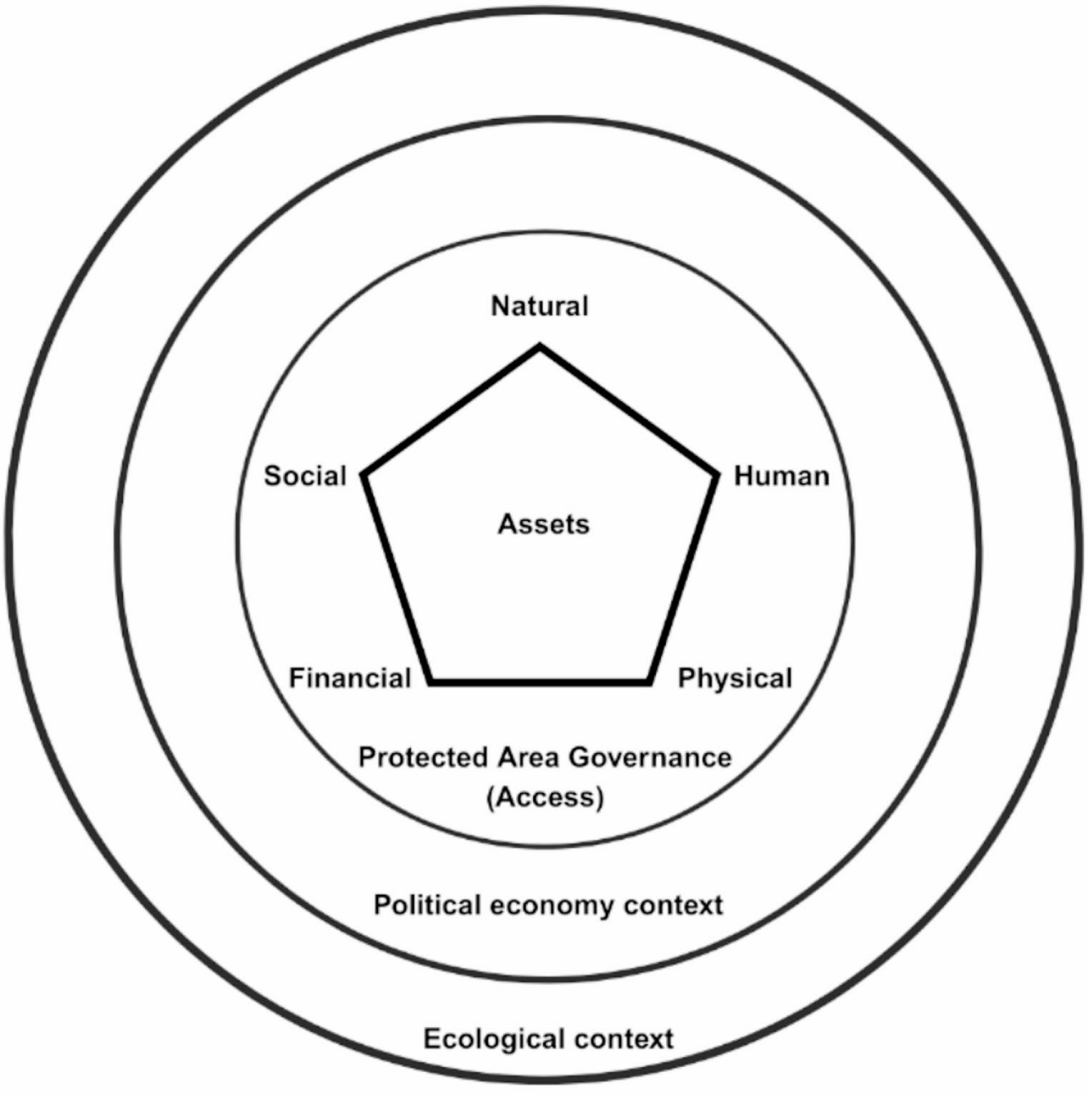
Theoretical framework visualization

The Sustainable Livelihoods framework provides a practical mechanism for integrating livelihood analysis with a political ecology perspective, as well as with access theory. It provides a more holistic measure of household assets and capabilities than econometric indices (Chambers & Conway, 1992; DFID, 1999; Scoones, 2009). Secondly, particular attention is paid to social capital as a means by which households can access influence, as well as other assets (Bebbington & Perreault, 1999; Ribot & Peluso, 2003). Thirdly, some analyses of the Sustainable Livelihoods framework include intangible assets as well as tangible assets, and this is a clear fit with the idea of influence as a critical, yet intangible, component of livelihoods and their governance (Chambers & Conway, 1992). Although commonly utilized and critiqued (Natarajan et al., 2022), the Sustainable Livelihoods Framework remains an important means of assessing household access to natural, human, social, physical, and financial assets, including in adapting to climate change (Pandey et al., 2017). The extent to which PA governance shapes access to assets via this framework has had limited attention paid to it. Figure 1 visualizes the study’s theoretical framework.

Based on the literature reviewed and this theoretical framework, two research questions were therefore posed in two Nepali PAs, one with a more a less centralized (ACA) and the other with a less centralized (SNP) governance model:

(1) What do household livelihoods involve, in terms of access to various asset classes, and which factors best explain them?
(2) How do household livelihoods vary between SNP and ACA, and to what extent does governance explain this variation, among other factors?

## Methods

### Study Areas

**Fig. 2:**
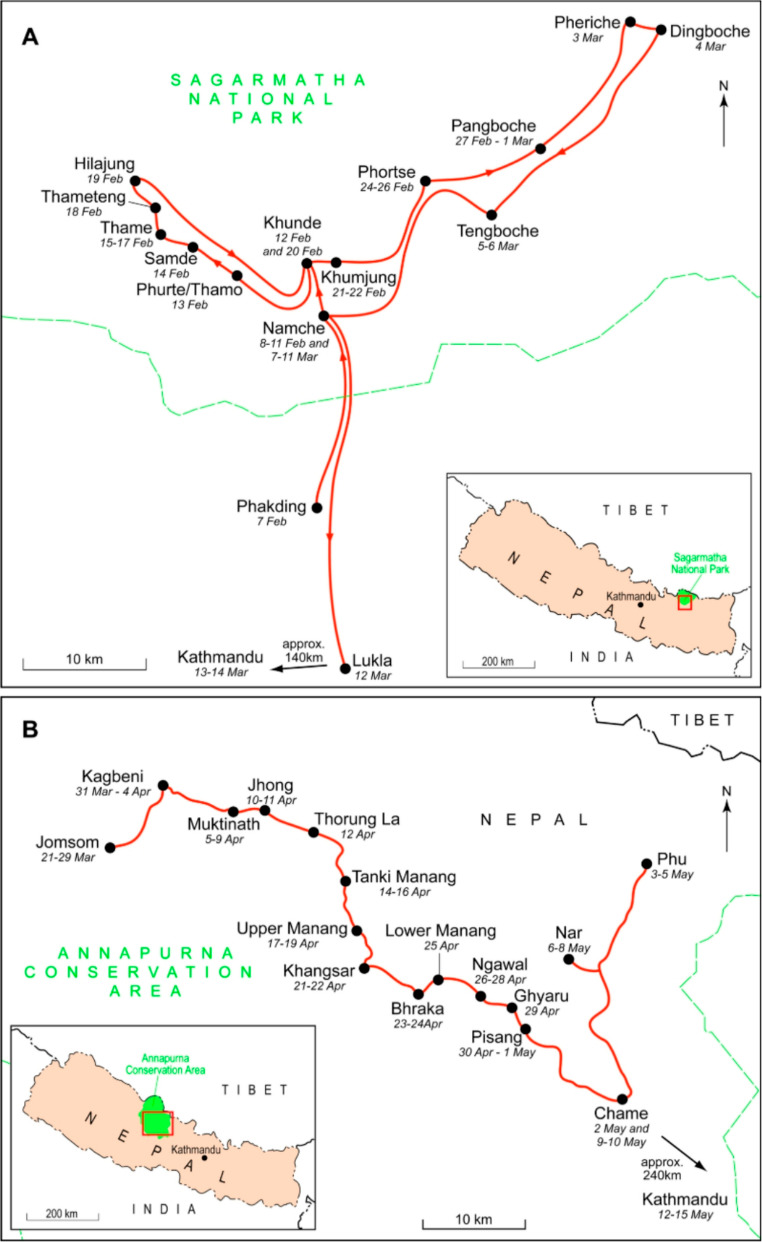
Study areas in Nepal showing areas and dates sampled in 2014. (**A**) Sagarmatha National Park. (**B**) Annapurna Conservation Area. Locations outside of study sites, and the dates visited, shown for illustrative purposes only

The ACA covers over 7,629 km² of Nepal’s mid-western region (Fig. 2), with elevation ranges between 1,000 m and 8,000 m. Iconic wildlife species include snow leopards (*Panthera uncia*) and blue sheep (*Pseudois nayaur)* (Bhuju et al., 2007). The PA is governed via a co-management approach, between Conservation Area Management Committees (CAMCs), drawn from local communities, and the National Trust for Nature Conservation (NTNC), a Nepali NGO. Of the c. 100,000 people who reside in the ACA (Government of Nepal, 2023), the majority derive their income from tourism and agro-pastoralism (Dangi et al., 2018). ACA is a popular trekking destination (Hanson et al., 2023) and received 181,000 international tourists in 2019, before declining sharply to 18,796 in 2020 due to the COVID-19 pandemic (ACAP, 2021).

Equally iconic, SNP (Fig. 2) was gazetted in 1976 (Baral & Heinen, 2005). Landscapes and habitat vary between permanent snow at 8,848 m and temperate forests at 2,845 m (Bhuju et al., 2007), with key wildlife species including snow leopard and Himalayan thar (*Hemitragus jemlahicus*). Approximately 7,000 people live within the park in 63 settlements (Government of Nepal, 2023), while in 2019 there were 57,000 visitors to the park (Bhatta et al., 2022). As with ACA, residents are engaged in a combination of agro-pastoral and tourism activities (Hanson, 2022). However, many of these practices are under pressure from climate change, among other environmental factors (Poudyal et al., 2021). SNP has a more centralized conservation governance regime, though with increasing local devolution since the introduction of a buffer zone in 2002 (Silwal et al., 2022), including a number of active grassroots NGOs and community organizations. This included the creation of Buffer Zone Management Committees and User Group Committees (Spoon, 2013).

## Questionnaire Preparation and Administration

Since family groups often pursue livelihood strategies, the household was the primary unit of analysis, a common approach in conservation social science assessments (Schreckenberg et al., 2010). Variables were initially chosen for the questionnaire based on a review of relevant literature (e.g., Bebbington & Perreault, 1999; Chambers & Conway, 1992; DFID, 1999; Scoones, 1998, 2009; Steimann, 2005) and were refined through a scoping trip to ACA in October/November 2013. For each category of the Sustainable Livelihoods Index, the specific indicators selected were based on a combination of scoping interviews and literature review, especially Steimann’s (2005) operationalization of the Index into a survey format from northwestern Pakistan, which was among the few available at the time. The goal was to include as broad and comprehensive a range of indicators as possible while keeping the household surveys reasonably short to prevent undue respondent fatigue (Glaser, 2012). To maximize respondent cooperation and based on insights from literature and scoping interviews, some potential variables, such as school and sanitation facilities, were excluded from the index design.

Two Nepali-speaking research assistants were recruited and trained to administer the household questionnaire, which was piloted with 24 households in the buffer zone surrounding SNP in February 2014. Questionnaire data were collected from 260 households in SNP between February and March 2014 (Fig. 2). In the ACA, 445 household questionnaires were completed between March and May 2014. Systematic sampling was used for the questionnaire due to the lack of a sampling frame for the settlements. The number of households in each Gaunpalika (formerly Village Development Committees [VDC]) was obtained from the 2011 census data (Government of Nepal, 2012), and a quarter of these were sampled, following Paudel and Thapa (2001). Each settlement was divided into two halves, with each research assistant collecting data in only one half. As recommended by White (2005), back-checking of 10% of household questionnaires was carried out by the Principal Investigator (PI).

Data were compared against 2011 census (Government of Nepal, 2012) rather than 2021 census (Government of Nepal, 2023) data for two reasons. Firstly, as Fieldwork occurred from February to May 2014, the 2011 census data allows for better comparisons temporally. Secondly, subsequent governance changes in Nepal in 2017 altered some administrative boundaries (Chaudhary, 2019), making like-for-like comparisons more challenging. Overall, the semi-historical nature of this data adds to other similar recent analysis from Nepal (Howlader & Ando, 2020; Nepal et al., 2020) to provide pertinent insights into current debates about PA governance and livelihoods nationally (Pandey et al., 2024), regionally (Chowdhury et al., 2022) and globally (Schleicher et al., 2019).

### Questionnaire Data Analysis

Following Steimann (2005), mean household access scores for each variable were computed. The results were also compared between the two study sites on a variable-by-variable basis using independent t-tests. The mean figures based on the five asset classes of the Sustainable Livelihoods framework were reviewed and aggregated for each class and overall, resulting in a Sustainable Livelihoods Index (SLI). While most variables recorded mean household access to a particular asset type, three were computed differently due to the characteristics of the data in question. Thus, the adult literacy rate was used as the educational access index. This is a practical and commonly accepted indicator of educational access, especially in contexts where more granular data is unavailable—such as many parts of South Asia and the broader Global South (Grotlüschen et al., 2020). Furthermore, it captures a broader range of education types, including informal, than measure of formal education alone (Bardsley et al., 2022). Similarly, the household livestock access index equaled household ownership or absence of each of the five livestock classes – cattle; sheep/goats; equines; yaks/yak/hybrids; other - with a minimum score of 0.00, a maximum score of 1.00 and intervals of 0.20. Thirdly, the financial assets index scores were allocated per household based on income class, with a minimum score of 0.167 for 0–50,000 Nepali rupees (NR), a maximum score of 1.00 for > 250,001, and intervals of 0.167. Those in the income class ‘prefer not to say’ were excluded from the index. The currency conversion rate at the time was 100 NR to 1 US dollar.

Independent t-tests were used successfully to test for inter-observer consistency between the two research assistants (Field, 2013). Quantitative data were analysed with descriptive and inferential statistics, with linear regression models being used for explanation rather than prediction (Mac Nally, 2000). Data were checked to ensure the necessary assumptions - linearity, reliability, homoscedasticity, and normality – for multiple regression models were met and that multicollinearity between variables did not exceed the recommended limit of 0.7 (Osborne & Waters, 2002). Instead of entry based on statistical significance alone, hierarchical entry based on theoretical suitability from the literature reviewed was used (Babulo et al., 2008; Block & Webb, 2001; Fasse & Grote, 2013; Parker & Thapa, 2012), with the r² change results utilized to determine goodness-of-fit used for model selection (Mac Nally, 2002). Bootstrapping was used for the final models as P-P plots to test for normality indicated some evidence of non-normality in the dependent variable (Field, 2013).

### Interviews

With a general structure mirroring that of the questionnaire, a semi-structured interview was also developed for the purposes of cross-methods, concurrent triangulation (Mikkelsen, 2005). It comprised mostly open questions but also gathered quantitative data on current market valuations of livestock. The interview sheet was piloted in ACA before commencement of the study. Snowball and convenience sampling were used to identify key local informants, with 26 interviews completed in SNP and 44 in ACA, representing 10% of the questionnaire sample.

Most interviews were administered in Nepali by two research assistants, with the PI always present. After transcription, the interview data were analysed both quantitatively and qualitatively to triangulate and complement the questionnaire data and analysis (Mikkelsen, 2005). This follows Nepal and Spiteri’s (2011) similar approach with their analysis of conservation and livelihoods in the Makalu-Barun Conservation Area, adjacent to SNP. Here, interview data are presented in the discussion, to complement and assist with the interpretation of the quantitative results.

### Results

Human assets included adult literacy, medicine, and media. Although the adult literacy rate was significantly higher in SNP than in ACA, overall medical and media access did not differ significantly between the two sites (Table 1). This resulted in a human assets index that was slightly higher in SNP (0.60 ± 0.19) than in ACA (0.57 ± 0.16) but not significantly so. The mean number of adults per household overall (N = 705) was 3.81 ± 1.90, with the figure of 3.98 ± 1.93 (N = 445) in ACA being significantly higher than the figure of 3.52 ± 1.81 (N = 260) in SNP (t (703)= −3.09; p = ≤ 0.05). Similarly, the mean number of people per household overall (N = 705) was 5.10 ± 2.10, with the figure of 5.28 ± 2.21 (N = 445) in ACA being significantly higher than the figure of 4.79 ± 1.87 (N = 260) in SNP (t (616)= −3.17; p = ≤ 0.05). At 0.97 ± 0.15 overall (N = 429), the school attendance rate did not vary significantly between SNP and ACA.

**Table 1:** Mean household human and natural assets access and indices

Indicator	N	Total ± SD	N	SNP ± SD	N	ACA ± SD	Difference
Educational access index (Adult literacy rate)	705	0.53 ± 0.32	260	0.59 ± 0.33	445	0.50 ± 0.30	t (503) = 3.32*
Self-administered traditional medicine	702	0.59 ± 0.49	257	0.49 ± 0.50	445	0.64 ± 0.48	*t* (515) = −4.11*
Self-administered ‘Western’ medicine	705	0.46 ± 0.50	260	0.60 ± 0.49	445	0.38 ± 0.49	*t* (703) = 5.55*
Visit to local clinic in Protected Area	705	1.00 ± 0.00	260	1.00	445	1.00	n/a
Visit to clinic outside of Protected Area	705	0.99 ± 0.11	260	0.97 ± 0.16	445	1.00 ± 0.05	*t* (285) = −2.39*
Medical access index	**702**	**0.76 ± 0.20**	**257**	**0.76 ± 0.22**	**445**	**0.76 ± 0.19**	***t*** **(482) = 0.36**
Newspaper	705	0.03 ± 0.17	260	0.00 ± 0.06	445	0.04 ± 0.20	*t* (573) = −3.76*
Radio	705	0.71 ± 0.46	260	0.70 ± 0.46	445	0.71 ± 0.45	*t* (703) = −0.52
Television	705	0.84 ± 0.37	260	0.78 ± 0.41	445	0.87 ± 0.34	*t* (466) = −2.66*
Internet	705	0.22 ± 0.41	260	0.28 ± 0.45	445	0.18 ± 0.39	*t* (477) = 3.06*
Media access index	**705**	**0.45 ± 0.21**	**260**	**0.44 ± 0.23**	**445**	**0.45 ± 0.19**	***t*** **(467) = −0.53**
**Human assets index**	**702**	**0.58 ± 0.17**	**257**	**0.60 ± 0.19**	**445**	**0.57 ± 0.16**	***t*** **(466) = 1.88**
Livestock access index	**705**	**0.33 ± 0.26**	**260**	**0.17 ± 0.15**	**445**	**0.43 ± 0.26**	***t*** (703) = −16.73*
Grazing land (index)	**705**	**0.84 ± 0.37**	**260**	**0.87 ± 0.33**	**445**	**0.82 ± 0.38**	***t*** **(603) = 1.84**
Cultivating land (index)	**705**	**0.94 ± 0.25**	**260**	**0.89 ± 0.32**	**445**	**0.96 ± 0.19**	***t*** (367) = −3.52*
Construction wood	705	0.92 ± 0.27	260	0.95 ± 0.22	445	0.90 ± 0.30	*t* (666) = 2.39*
Human food	704	0.95 ± 0.22	259	0.98 ± 0.12	445	0.93 ± 0.25	*t* (686) = 3.67*
Animal food	699	0.96 ± 0.20	254	0.96 ± 0.19	445	0.95 ± 0.21	*t* (697) = 0.74
Medicinal plants	700	0.79 ± 0.41	225	0.69 ± 0.46	445	0.84 ± 0.36	*t* (435) = − 0.4.52*
Natural products access index	**699**	**0.91 ± 0.16**	**254**	**0.90 ± 0.15**	**445**	**0.91 ± 0.17**	***t*** **(697) = −0.40**
Spring	705	0.27 ± 0.44	260	0.23 ± 0.42	445	0.29 ± 0.46	*t* (578) = −1.93
Well	705	0.00 ± 0.07	260	0.01 ± 0.11	445	0.00 ± 0.00	*t* (259) = 1.74
Handpump	705	0.00 ± 0.05	260	0.01 ± 0.09	445	0.00 ± 0.00	*t* (259) = 1.42
Outside tap	705	0.99 ± 0.11	260	0.97 ± 0.17	445	1.00 ± 0.05	*t* (282) = −2.60*
Inside tap	705	0.44 ± 0.50	260	0.37 ± 0.48	445	0.48 ± 0.50	*t* (558) = −2.97*
Water access index	**705**	**0.34 ± 0.11**	**260**	**0.32 ± 0.13**	**445**	**0.35 ± 0.10**	***t*** (442) = −3.97*
**Natural assets index**	**699**	**0.67 ± 0.13**	**254**	**0.63 ± 0.14**	**445**	**0.69 ± 0.13**	***t*** (697) = −5.91*

* p = ≤ 0.05. Indices in bold. Educational access index equals adult literacy rate. Medical and media access indices equal means of respective preceding variables. Human assets index equals mean of educational, medical and media access indices. Livestock access index equals household ownership or absence of each of the five livestock classes – cattle; sheep/goats; equines; yaks/yak/hybrids; other - with a minimum score of 0.00, a maximum score of 1.00 and intervals of 0.20

Natural assets included livestock, land for grazing and cultivation, natural products, and water. Livestock access was significantly higher in ACA, despite access to grazing land being slightly lower than in SNP (Table 1). However, a significantly higher proportion of households were able to access land for cultivation in ACA (Table 1). Access to natural products did not differ significantly between the two sites but water access was significantly higher in ACA (Table 1). Mean livestock ownership per household overall (N = 705) was 15.48 ± 30.53, with the figure of 5.43 ± 9.04 (N = 260) in SNP being significantly lower than the figure of 21.35 ± 36.56 (N = 445) in ACA (t (532)= −8.74; p = ≤ 0.05). In terms of horticultural production, a significantly higher (t (460) = −5.09; p = ≤ 0.05) proportion of households in ACA (N = 430; 0.63 ± 0.48) sold surplus crops compared to SNP (N = 231; 0.43 ± 0.50). The natural asset index score was significantly higher in ACA (Table 1).

Social assets included social organizations and political representation. While overall access to organizations was not significantly different between the two study sites (Table 2), significant differences were observed with involvement in specific types of organizations. Access to microcredit and co-operative groups were significantly higher in SNP, while access to women’s groups and school associations (also known as School Management Committees – see Puri & Chhetri, 2024) were significantly higher in ACA. Similarly, while political access was fairly uniform across both sites, access to local political representation was significantly higher in ACA and access to national-level political representation was significantly higher in SNP. Overall, the social assets index scores were identical across both locations (Table 2).

**Table 2:** Mean household social, physical, and financial assets access and indices

Indicator	N	Total ± SD	N	SNP ± SD	N	ACA ± SD	Difference
Conservation committee	701	0.12 ± 0.32	256	0.15 ± 0.36	445	0.10 ± 0.30	*t* (464) = 1.79
Village development (from 2017, Gaunpalika) committee	702	0.05 ± 0.22	257	0.06 ± 0.24	445	0.04 ± 0.20	*t* (700) = 0.93
Tourism association	702	0.10 ± 0.30	257	0.11 ± 0.31	445	0.09 ± 0.29	*t* (700) = 0.72
Microcredit group	702	0.03 ± 0.17	257	0.06 ± 0.24	445	0.01 ± 0.09	*t* (302) = 3.38*
Producer/agricultural co-operative	702	0.06 ± 0.24	257	0.15 ± 0.36	445	0.01 ± 0.11	*t* (282) = 6.01*
Women’s group	703	0.51 ± 0.50	258	0.28 ± 0.45	445	0.64 ± 0.48	*t* (567) = −10.29*
School association/School management committee	702	0.09 ± 0.29	257	0.06 ± 0.24	445	0.11 ± 0.32	*t* (657) = −2.58*
Youth group	703	0.40 ± 0.49	258	0.38 ± 0.49	445	0.41 ± 0.49	*t* (544) = −0.98
Other	702	0.08 ± 0.27	257	0.09 ± 0.28	445	0.07 ± 0.26	*t* (700) = 0.66
Organizational access index	**701**	**0.16 ± 0.15**	**256**	**0.15 ± 0.17**	**445**	**0.17 ± 0.14**	***t*** **(448) = −1.67**
Local elected representatives	704	0.98 ± 0.15	259	0.94 ± 0.23	445	1.00 ± 0.00	*t* (258) = −3.98*
District elected representatives	704	0.66 ± 0.48	259	0.69 ± 0.47	445	0.64 ± 0.48	*t* (555) = 1.33
National elected representatives	704	0.06 ± 0.23	259	0.12 ± 0.32	445	0.02 ± 0.16	*t* (330) = 4.29*
Political access index	**704**	**0.56 ± 0.20**	**259**	**0.58 ± 0.25**	**445**	**0.55 ± 0.17**	***t*** **(410) = 1.65**
**Social assets index**	**701**	**0.36 ± 0.14**	**256**	**0.36 ± 0.17**	**445**	**0.36 ± 0.12**	***t*** **(408) = 0.27**
Fuelwood	705	0.96 ± 0.19	260	0.94 ± 0.24	445	0.98 ± 0.14	*t* (365) = −2.53*
Cylinder gas	705	0.61 ± 0.49	260	0.40 ± 0.49	445	0.74 ± 0.44	*t* (496) = −9.02*
Kerosene oil	705	0.37 ± 0.48	260	0.58 ± 0.49	445	0.25 ± 0.44	*t* (489) = 8.84*
Electricity	705	0.79 ± 0.41	260	0.95 ± 0.23	445	0.70 ± 0.46	*t* (686) = 9.62*
Animal dung	705	0.60 ± 0.49	260	0.90 ± 0.31	445	0.42 ± 0.50	*t* (701) = 15.63*
Other	705	0.05 ± 0.21	260	0.08 ± 0.27	445	0.03 ± 0.16	*t* (373) = 2.74*
Fuel access index	**705**	**0.56 ± 0.17**	**260**	**0.64 ± 0.15**	**445**	**0.52 ± 0.17**	***t*** (703) = 9.38*
Residential building	705	0.76 ± 0.43	260	0.62 ± 0.49	445	0.85 ± 0.36	*t* (423) = − 0.6.75*
Joint tourist/residential building	705	0.24 ± 0.43	260	0.35 ± 0.48	445	0.17 ± 0.38	*t* (447) = 5.18*
Tourist building	705	0.02 ± 0.13	260	0.02 ± 0.15	445	0.01 ± 0.12	*t* (703) = 0.95
Other	705	0.09 ± 0.29	260	0.12 ± 0.33	445	0.07 ± 0.26	*t* (451) = 2.05*
Building access index	**705**	**0.28 ± 0.08**	**260**	**0.28 ± 0.08**	**445**	**0.28 ± 0.08**	***t*** **(703) = 0.14**
Foot	705	1.00 ± 0.07	260	1.00 ± 0.00	445	0.99 ± 0.08	*t* (444) = 1.74
Animal	705	0.47 ± 0.50	260	0.53 ± 0.50	445	0.44 ± 0.50	*t* (703) = 2.54*
Bicycle	705	0.01 ± 0.09	260	0.00 ± 0.0	445	0.01 ± 0.12	*t* (444) = −2.46*
Bus/taxi	705	0.51 ± 0.50	260	0.04 ± 0.19	445	0.78 ± 0.41	*t* (676) = −32.59*
Airplane	705	0.51 ± 0.50	260	0.89 ± 0.31	445	0.28 ± 0.45	*t* (684) = 21.27*
Motorcycle	705	0.21 ± 0.41	260	0.01 ± 0.09	445	0.33 ± 0.47	*t* (495) = −13.90*
Other	705	0.03 ± 0.17	260	0.07 ± 0.26	445	0.00 ± 0.05	*t* (269) = 4.34*
Transport access index	**705**	**0.39 ± 0.13**	**260**	**0.36 ± 0.10**	**445**	**0.41 ± 0.15**	***t*** (678) = −4.37*
**Physical assets index**	**705**	**0.41 ± 0.08**	**260**	**0.43 ± 0.07**	**445**	**0.40 ± 0.08**	***t*** (619) = 4.47*
**Financial assets index**	**608**	**0.69 ± 0.32**	**238**	**0.65 ± 0.3**	**370**	**0.71 ± 0.31**	***t*** **(606)** = −2.02*

* p = ≤ 0.05. Indices in bold. Organizational and political access indices equal means of respective preceding variables. Social assets index equals mean of preceding social indices. Fuel, building and transport access indices equal means of respective preceding variables. Physical assets index equals mean of preceding fuel, building and transport access indices. Financial assets index scores were allocated per household based on income class, with a minimum score of 0.167 for 0–50,000, a maximum score of 1.00 for > 250,001, and intervals of 0.167. Those in the income class ‘prefer not to say’ were excluded from the index. Currency conversion rate: 100 Nepali rupees to 1 US dollar

Physical assets included fuel, buildings, and transport. Access to overall fuel types was significantly higher in SNP than in ACA (Table 2). Of these, access to fuelwood and cylinder gas was significantly higher in ACA, while access to kerosene oil, electricity, animal dung and other sources were significantly higher in SNP. Access to buildings was equal overall between the two sites, as was access to buildings specifically for tourism (Table 2). In ACA, access to residential buildings was significantly higher than in SNP, but access to other types of buildings, as well as buildings for joint tourism-residential use, were significantly lower. Of the two areas, ACA had significantly better transport connections overall (Table 2). This included bicycle, bus/taxi, and motorcycle, but not animals, airplanes or other. In total, access to physical assets was significantly higher in SNP (Table 2).

For household financial income (N = 705), 11.1% claimed an annual financial income of US$ 0–50; 13.8% US$ 51–100; 8.9% US$ 101–150; 9.8% US$ 151–200; 6.0% US$ 201–250; and 36.7% >US$ 250. 13.8% of respondents preferred not to report their annual financial income and were discounted from further financial assets, or SLI, calculations. Of the remaining 608 households, mean annual financial income was significantly higher in ACA (Table 2). Cultivation (0.83 ± 0.38), followed by livestock herding (0.56 ± 0.50) and then tourism (0.42 ± 0.49) were the most accessed sources of financial income (N = 705). When listed as priorities (N = 705), agriculture was cited as the most important (43.4%), followed by tourism (31.5%), other (14.2%), livestock (6.7%) and remittances (4.2%).

**Fig. 3:**
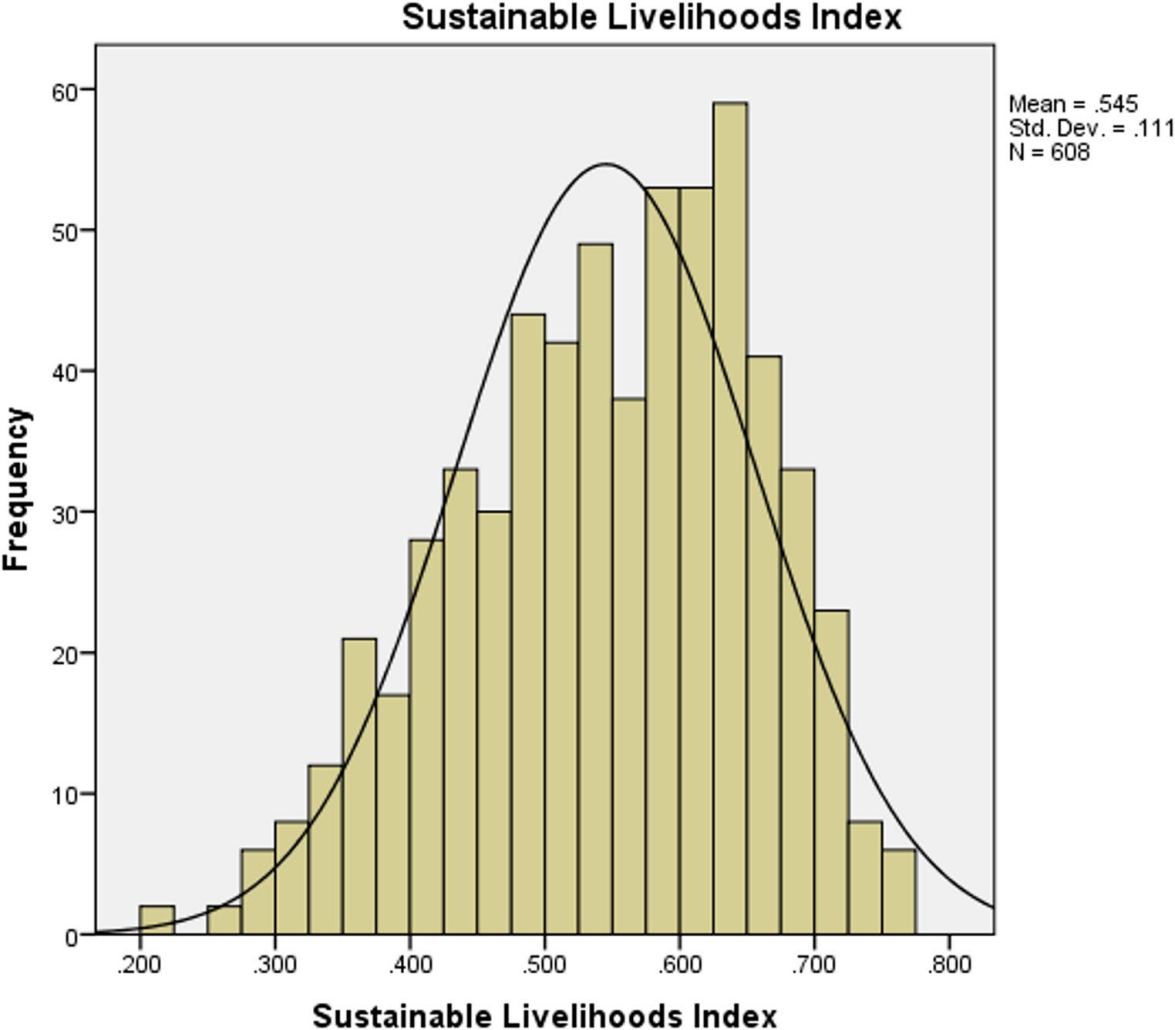
Sustainable livelihoods index

The mean SLI score for the combined sample was 0.54 ± 0.11, with a median of 0.55, a minimum of 0.21 and a maximum of 0.77 (Table 3; Fig. 3). The SLI scores were significantly higher in ACA than in SNP (Table 3). Contributing to this, despite significantly higher levels of access to physical assets in SNP, were significantly higher levels of access to natural and financial assets in ACA. Access to human and social assets did not differ significantly between the two sites

**Table 3:** Mean household livelihoods asset access indices

Index	N	Total ± SD	N	SNP ± SD	N	ACA ± SD	Difference
Human assets	702	0.58 ± 0.17	257	0.60 ± 0.19	445	0.57 ± 0.16	*t* (466) = 1.88
Natural assets	699	0.67 ± 0.13	254	0.63 ± 0.14	445	0.69 ± 0.13	*t* (697) = −5.91*
Social assets	701	0.36 ± 0.14	256	0.36 ± 0.17	445	0.36 ± 0.12	*t* (408) = 0.27
Physical assets	705	0.41 ± 0.08	260	0.43 ± 0.07	445	0.40 ± 0.08	*t* (619) = 4.47*
Financial assets	608	0.69 ± 0.32	238	0.65 ± 0.33	370	0.71 ± 0.31	*t* (606) = −2.02*
Sustainable Livelihoods Index	**608**	**0.54 ± 0.11**	**238**	**0.53 ± 0.12**	**370**	**0.55 ± 0.10**	***t*** (449) = −2.42*

* p = ≤ 0.05. Sustainable livelihoods index equals mean of indices from each of the five asset classes

.

Of the linear regression models explaining household SLI scores, the ACA model explained 62.2% of variance, followed by the SNP model at 36.7% and the combined model with 32.0% (Table 4). Income from tourism was the most important factor across all three models, followed by household size. In ACA, but not SNP, income from livestock made a notable contribution to the model. Other independent variables significant in all three models included income from savings and/or remittances. Income from cultivation was not significant in any of the models.

**Table 4:** Linear regression models explaining household sustainable livelihoods index scores for a combined sample, in SNP and in ACA

Model	Variable	b	SE B	Standardized b	p
Combined R² = 0.320 N = 608	**Constant**	0.34 (0.31, 0.37)	0.015	---	*p* =.001
	**Household size**	0.013 (0.009, 0.017)	0.002	0.25	*p* =.001
	Savings income*	0.054 (0.028, 0.081)	0.013	0.13	*p* =.001
	Remittance income*	0.031 (0.010, 0.051)	0.010	0.11	*p* =.001
	Livestock income*	0.027 (0.009, 0.044)	0.008	0.12	*p* =.003
	Cultivation income*	0.021 (−0.002, 0.045)	0.012	0.071	*p* =.093
	Tourism income*	0.12 (0.11, 0.14)	0.009	0.55	*p* =.001
SNP R² = 0.367 N = 238	**Constant**	0.37 (0.32, 0.41)	0.019	---	*p* =.001
	**Household size**	0.013 (0.007, 0.019)	0.003	0.20	*p* =.001
	Savings income*	0.068 (0.027, 0.11)	0.020	0.18	*p* =.002
	Cultivation income*	0.021 (−0.008, 0.053)	0.015	0.081	*p* =.15
	Tourism income*	0.12 (0.093, 0.15)	0.014	0.45	*p* =.001
ACA R² = 0.622 N = 370	**Constant**	0.39 (0.35, 0.44)	0.020	---	*p* =.001
	**Household size**	0.013 (0.008, 0.018)	0.002	0.28	*p* =.001
	Remittance income*	0.025 (0.003, 0.048)	0.010	0.10	*p* =.019
	Livestock income*	0.044 (0.027, 0.060)	0.009	0.21	*p* =.001
	Cultivation income*	0.026 (−0.011, 0.060)	0.019	0.068	*p* =.16
	Tourism income*	0.12 (0.099, 0.13)	0.010	0.50	*p* =.001

* = 0 = no; 1 = yes

## Discussion

### Human Assets

The mean household size of 3.81 adults was a similar figure to that of 3.44 from the 15 relevant Gaunpalikas (formerly VDCs) in the 2011 Nepal Census (Government of Nepal, 2012). As in other areas of the Nepal Himalaya, some households may temporarily increase in size, from additional family and other informal or casual forms of labor, due to the seasonal demands of the tourist trade (Hanson et al., 2023; Nepal, 2000). The combined adult literacy rate of 53% was somewhat lower than the national average of 59.6% (Government of Nepal, 2012), though the significantly higher rate in SNP equaled the national average. This may reflect the significant educational, and other forms of, investment made in the area by mountaineering related-philanthropy from 1953 onwards (McDowell et al., 2013; Silwal et al., 2022). By contrast, the NTNC has only been active in ACA, including in education, since 1986 (Bhuju et al., 2007). The proportion of children attending school reported in questionnaire responses – 97% - was similar to results derived from triangulation interviews, where 98.6% and 87.1% of children attended primary and secondary school, respectively.

Data on medical access showed there was universal access to clinics within the PAs and almost universal access outside PAs, though with lower rates of traditional medicine—known as *amchi*—usage. Triangulation interviews confirmed widespread access to clinics within the PAs but indicated higher rates of traditional medicine use than in the questionnaire, although they noted that its availability and importance was decreasing, particularly among younger generations (Conservation leader and teacher, SNP; Tourism officer, ACA; Health worker, ACA; Conservation leader x2, ACA). Media access was mostly influenced by mountain logistics, where geographic isolation and rugged terrain in mountain regions can significantly limit access to media and communication technologies (Dax, 2022). Newspaper usage was nearly nonexistent, it being concentrated in more urban and low-lying areas of the country (Ojha & Kumar, 2022), but there were relatively high access rates to radio and television, along with increasing internet access. Overall, although a decentralized approach might be expected to improve access to education and healthcare, the lack of a significant overall difference in human assets between ACA and SNP may be due to other sources of investment in the latter, including mountaineering-related philanthropy and overseas development assistance (ODA), which could offset community-NGO spending in ACA (McDowell et al., 2013; Silwal et al., 2022).

### Natural Assets

Pastoralism and agro-pastoralism continue to play an important role in the Hindu-Kush Himalayan region (Sandhu & Sandhu, 2015), and pastoralism continues to play an important role in both ACA and SNP, though more so in the former. The significantly higher mean livestock owned per household in ACA is similar to previous figures of 26.6 reported from Manang (Oli et al., 1994) and of 18.1 reported from Upper Mustang (Aryal et al., 2014). Various factors may explain these data. Sheep and goats have gradually been eliminated from SNP to reduce deforestation (Bhuju et al., 2007; Hanson, 2022). This may have only been possible under a centralized conservation governance model, where the PA authorities have the power necessary to dictate overall policy objectives. Equines, principally used for transport, have long been associated with the trading routes that pass through ACA to and from Tibet (Wright, 2015). Yaks and yak hybrids may be equally present at both sites due to their cultural significance (Teacher, ACA; Conservation leader, ACA). There were also contrasting trends in livestock production in SNP and ACA. More livestock farmers engaged in semi-commercial or commercial production in SNP (Table S1), despite the livestock extension program of the NTNC in ACA (Dangi et al., 2018). Several interviewees commented that the greater commercialization seen in SNP may be a by-product of the transportation for the tourism industry (Conservation leader x 2, SNP), a trend also noted by Hanson et al. (2023).

More widespread access to agricultural land in ACA may be due to the larger land area of this PA. In keeping with this result, surplus crops were sold more often in ACA than in SNP, a factor that triangulation of interview data corroborated (Table S1). However, fewer respondents than for livestock production felt that agricultural production was decreasing and the range of reasons provided for this trend was broadly similar at both sites, although none mentioned switching to cash crops in SNP (Table S1). The role of the NTNC in providing an agricultural extension service in ACA (Aase et al., 2013; Dangi et al., 2018), including the development of social capital, as well as easier market access via roads, may explain why agriculture is more commercialized in ACA than in SNP.

Various natural products were readily available to households in both ACA and SNP. Sources of water were mostly equally accessible across both PAs, with the exceptions of outside and inside water taps. Levels of usage of wells and hand-pumps were either non-existent for both categories in SNP or extremely low in ACA, reflecting the greater importance of gravity-fed natural water systems at both sites, a trend common across the high mountain ecosystems in Nepal (Sharma et al., 2005). The greater availability of taps in ACA is probably linked to NTNC community development programs (Nepal et al., 2022). As a result, the water access index scores were significantly higher in ACA than in SNP, reflecting also the increasing pressure being put on water supplies in SNP by climate-related hydrological changes (Poudyal et al., 2021). Overall, the larger area and greater variety of landscapes in ACA may explain why natural assets are more widely available there. Equally, access to these assets is mediated by a devolved governance regime, including the supportive role of the NTNC, which has generally been more tailored to participatory and sustainable use in ACA than in SNP.

### Social Assets

Despite its importance in the Sustainable Livelihoods framework (Natarajan et al., 2022; Steimann, 2005) and access theory (Ribot, 2014; Ribot & Peluso, 2003), social capital has remained difficult to quantify in household surveys. Access to organizations is one measure, of which there were a wide range of options. Membership of women and youth organizations was most common, with approximately one in eight households having membership of a conservation committee, whether a formal Conservation Area Management Committee (CAMC) in ACA (Bajracharya et al., 2006) or other forms of conservation committee in SNP, such as those related to the management of the buffer zone (Spoon, 2013). Triangulation interviews also identified that these three were the organizations in which households were most frequently involved. Household membership of various types of organizations showed that ACA scored slightly higher on this index, but any differences were small. This is surprising given the decentralized governance model in ACA, and the investment in co-management and social capital made by the NTNC and local communities (Baral et al., 2007; Baral & Stern, 2011; Nepal et al., 2022). However, organizational membership is not necessarily synonymous with devolved governance, and despite greater devolution to three buffer zone user committees in SNP from 2002 onwards (Silwal et al., 2022), the active control of conservation and development is still not as comprehensively community-based as in ACA.

Political access is another measure of social capital that it is mediated by geographical distance. In this study, national representation in Kathmandu was the most difficult to access. This compares with the political marginalization of mountain regions observed elsewhere (Ellis-Jones, 1999) and was corroborated by triangulation interviews. Interviewees also noted the importance of wealth and power in accessing political representation (Microcredit cooperative officer, SNP), an important component of access theory (Ribot & Peluso, 2003). Household access to political representation in ACA and SNP showed no difference overall, although this masks significantly better access to local elected representatives in ACA, and significantly better access to national-level elected representatives in SNP. Easier local access in ACA may be due to the smaller Gaunpalikas (formerly VDCs) in Manang and Mustang districts (Government of Nepal, 2012). Better national access in SNP could be explained by the large number of Sherpa hoteliers living in Kathmandu on a permanent or seasonal basis (Park officer, SNP.), or by the direct flights between Solukhumbu district and Kathmandu.

### Physical Assets

Access to physical assets, such as firewood, continues to be an important source of energy for households, followed by electricity, cylinder gas and animal dung. These four energy sources were also the most mentioned in triangulation interviews. The significance of fuelwood for households echoes similar findings in Nepal generally (Howlader & Ando, 2020). Fuel types were more accessible in SNP than in ACA, except for fuelwood and cylinder gas. Even though the NTNC has organized alternative energy and energy efficiency programs in ACA (Dangi et al., 2018; Nepal et al., 2022), SNP has benefited from significant investment in numerous micro-hydro schemes (Park officer, SNP). As with medical and educational spending, this may be largely funded by mountaineering-related philanthropy and development assistance, such as via the Swiss and New Zealand governments (McDowell et al., 2013; Silwal et al., 2022).

Given that only 26% of households had access to either a joint tourist/residential building or a tourist building, it would suggest that the tourist industry in ACA and SNP is controlled by a minority of the population, a concern raised by several researchers in SNP (Daconto & Sherpa, L. N., 2010; Nepal, 2000). Similarly, while households in ACA had significantly more access to residential buildings, households in SNP had significantly more access to joint tourist/residential buildings. This would seem to support suggestions that residents in SNP are more involved in the tourism industry than in ACA (Nepal et al., 2022; Silwal et al., 2022), despite significant NTNC-supported development of tourism activities in the latter.

Access to transport is largely a product of the differing logistical scenarios at each site. SNP is served by a well-established airport in the Solukhumbu district, which is used by many tourists and locals to access the main trekking routes and settlements (Bhuju et al., 2007). This explains the significantly greater access to transport by airplane and other means – including helicopter – in SNP. As air transport is not routinely used for bulk freight, it also explains the greater use of animals for transport here. In contrast, the traditional trekking routes into and around ACA did not necessitate air transport, although there are now motorable roads into both Manang and Mustang districts (Dangi et al., 2018). This explains the greater access to bus/taxi and motorcycle transport in ACA but, as indicated by a number of interviewees (Teacher, ACA; Hotelier and community leader, ACA), this has resulted in a decreased use of animal transport. Road access contributes to the significantly higher transport access index scores in ACA. However, when all three physical assets indices are aggregated, SNP has better access overall to physical assets, but this is largely a product of factors other than governance, such as infrastructure.

### Financial Assets

Questionnaire data on mean household incomes showed that many respondents were earning more than NR 250,001 (USD 2,500) per annum. This figure suggests that earnings were higher than anticipated and probably indicates that the distribution of wealth is weighted towards those households who can effectively access and control the lucrative tourist trade in both PAs. Such concerns are of particular importance in fragile mountain ecosystems like the Nepal Himalaya, where they can disrupt longstanding social-ecological structures and customs, and have been raised previously in SNP (Bhatta et al., 2022; Daconto & Sherpa, 2010; Nepal, 2000). In contrast, the significantly higher financial assets index scores in ACA may be attributed, at least in part, to the greater involvement of the community in conservation and development activities, as well as the supporting role of the NTNC (Aase et al., 2013; Dangi et al., 2018; Nepal et al., 2022).

Cultivation, followed by livestock herding and then tourism, was the most accessed source of financial income. As ranked income priorities, agriculture and tourism were the most important activities, with livestock herding being much less so, indicating pastoralism may provide a constant, low level of income. Agro-pastoralism continues to be significant livelihood for communities here, as well as across the region (Aase et al., 2013; Sandhu & Sandhu, 2015; Hanson, 2022). However, the role of the NTNC in supporting livelihood diversification and innovation in ACA (Aase et al., 2013; Dangi et al., 2018) may have made households there less dependent on tourism alone than in SNP.

### Sustainable Livelihoods Index Scores

The creation of the SLI followed the asset classes of the Sustainable Livelihoods framework (Chambers & Conway, 1992; DFID, 1999; Scoones, 2009), and particularly of Steimann’s (2005) application of them in questionnaire format in Pakistan. As a measure of livelihood diversification and resilience, the overall SLI scores comprised higher levels of human, natural and financial assets, and lower levels of social and physical assets. Particularly high levels of income amongst a large proportion of households may have contributed to the skewed distribution curve (Fig. 3). Elsewhere, households with more assets tend to be more resilient to risks (Pandey et al., 2017; Salerno et al., 2015).

Although SLI scores were significantly higher for ACA than for SNP, it is not possible to attribute causation to these differences on the basis of governance model alone, as a comparison of livelihoods and wildlife impacts in and around CBC areas in northern Tanzania also noted (Salerno et al., 2015). However, it is likely that the decentralized management model in ACA does contribute to its higher SLI scores. The gap between the two is less than expected, which is likely due to the 2002 policy change which saw the creation of buffer zone user groups in SNP (Budhathoki, 2004; Silwal et al., 2022), as well as to a heritage of mountaineering-related philanthropy and ODA in the area since the 1950 s (McDowell et al., 2013).

However, broadly similar access to these assets across both PAs is different from access to the increased influence which co-management/decentralization ostensibly brings. The role of the NTNC is particularly noteworthy, as part of such an innovation system in ACA that also includes local communities, the Government of Nepal and international agencies and donors (Aase et al., 2013; Dangi et al., 2018; Nepal et al., 2022). In addition, access to assets, and undoubtedly access to influence in its various forms also, is lacking more amongst female and lower-caste park residents (Baral et al., 2007). However, an assessment of this was beyond the scope of this study because the household, rather than the individual, was the main unit of analysis.

### Explaining Sustainable Livelihoods Index Scores

In multivariate analysis, income from tourism was the most influential explanatory factor. Given that tens of thousands of, mostly foreign, tourists visit SNP and ACA each year (ACAP, 2021; Baral et al., 2007; Hanson et al., 2023), it is no surprise that access to this lucrative industry can contribute to livelihood improvements and sustainability, particularly in terms of financial income. Forms of financial income from the 42% of households who accessed the tourism industry included via the provision of accommodation, guiding, portering and transportation, as well as sales of food and equipment (Nepal et al., 2022; Silwal et al., 2022). However, as has been noted with PAs in East Africa (Munanura et al., 2016; Sandbrook & Adams, 2012), concerns persist about the equitable distribution of benefits from tourism within both ACA and SNP (Nepal, 2000), as well as the increasing market share of external business interests, both national and international (Daconto & Sherpa, 2010). The greater contribution of tourism to household incomes in SNP than in ACA is unsurprising and consistent with the literature (Bajracharya et al., 2006). In part, this may be due to the investment of the NTNC, alongside local CAMCs and Gaunpalikas (formerly VDCs), in forms of livelihood creation other than tourism (Aase et al., 2013; Dangi et al., 2018; Nepal et al., 2022), something that the less decentralized SNP lacks to the same extent.

Household size was also an important explanatory factor in the multiple regression model. From the perspective of a Sustainable Livelihoods framework perspective, this may be explained in terms of the quantity of human assets available to engage in livelihood creation (Berzborn, 2007). More household members may equate to employing a more diverse range of livelihood strategies, a trend also observed in an assessment of livelihood diversification predictors in Ethiopia (Block & Webb, 2001). It may also interact with these other factors by providing additional family members to be involved in additional income generating activities; it is common in Nepal for men to work in paid employment outside of the home, region, or country, while women, children and other dependents maintain the home, farm, and other local activities (Sunam & McCarthy, 2016).

After tourism and household members, the two models then diverged in their similarities. Livestock, for instance, were found to be a significant contributor to higher SLI scores in ACA but not SNP. Part of this can undoubtedly be explained due to higher mean livestock ownership per household in ACA, a trend which is itself partly explained by the elimination of sheep and goats from SNP for conservation reasons (Bhuju et al., 2007; Silwal et al., 2022). An additional factor may be enhanced access to ACA via roads into both Manang and Mustang districts, something altogether lacking in SNP. Livestock extension services have been provided by the NTNC, in partnership with local CAMCs and Gaunpalikas (formerly VDCs) (Aase et al., 2013; Bajracharya et al., 2006; Dangi et al., 2018). In this sense, co-management of this PA may therefore have contributed directly to livestock being a significant and important income source for households in ACA. Even though only 6.67% of households identified livestock as their most important source of income, this positive relationship to household SLI scores in ACA demonstrates the importance of livestock as an asset bank and income source (Devendra & Chantalakhana, 2002; Velho et al., 2019).

Access to financial income from various sources was the next-most significant factor in explaining higher SLI scores at both sites. However, the precise nature of this source differed, as income from savings was an important contributor in SNP, while income from remittances was important in ACA. Triangulation interviews also confirmed these findings: remittances were mentioned more frequently as an income source in ACA than in SNP, and savings were mentioned in SNP and not in ACA. Access to financial capital is associated with more sustainable, diverse livelihoods (Block & Webb, 2001), and in SNP can be partly credited to significantly higher microcredit group membership.

### Protected Area Governance and Livelihoods

There are broader lessons about PA governance and livelihoods that can be drawn from this comparative study. In relation to SNP and ACA, their theoretically distinct governance models have converged to a considerable extent in practice due to policy and legislative changes surrounding PA management in Nepal, especially the creation of a buffer zone and its user groups from 2002 (Aryal et al., 2021; Nepal et al., 2022; Velho et al., 2019). Nevertheless, the sites remain high-profile outliers due to the scope and scale of tourism development (Bhatta et al., 2022; Nepal et al., 2022), as well as the sustained investment in conservation and development by multilateral, ODA, NGO, and philanthropic sources that have improved livelihoods across the SLI dimensions.

Secondly, beyond the two study sites, between 2015 and 2017 Nepal underwent constitutional reforms aimed at decentralizing authority via a more federal structure (Chaudhary, 2019). These reforms introduced stronger local governance frameworks, intended to reshape how power, resource access, and decision-making responsibilities are distributed at the subnational level. The consequences of these changes for PAs and their governance are still becoming apparent. Nonetheless, concerns have been raised that the newly formed provincial and local government bodies may, paradoxically, centralize control, potentially weakening the participatory and community-led governance systems that had previously guided much co-management of PAs and natural resources (Thakali et al., 2018). As Thakali et al. (2018) point out, not only would this be deeply ironic, but it may threaten the contribution of these bottom-up approaches and institutions to Nepal’s ongoing work in conservation and development. They recommend greater conservation governance-focused capacity building and funding to ensure the newer governance structures complement and expand upon existing efforts. The findings from this study suggest that the investments made in the long-term creation of conservation governance social capital have important implications for local livelihoods and are worth maintaining and even expanding.

Thirdly, beyond Nepal and in the context of proposals to protect half of the world’s surface area for biodiversity (Dinerstein et al., 2017), the issues of PA governance and livelihood impacts remain significant (Schleicher et al., 2019). As the examples of SNP and ACA demonstrate, different approaches to PA governance will continue to exist, alongside the unique social, ecological, and economic characteristics of the world’s PA network. Nevertheless, this study illustrates not only that developing social capital, via participatory institutions, can play an important role in more decentralized parks like ACA, but also that, through policy and legislative changes like buffer zone user groups, these approaches can also be retrofitted to more centralized PAs like SNP.

## Conclusion

That enhanced access to assets - especially household members, tourism income and, in the case of ACA, livestock - should improve livelihoods and, in turn, reproduce access to further assets is consistent with the theory of access (Ribot & Peluso, 2003). Although both the theoretical framework and the broader literature suggest that a more decentralized approach to PA governance would contribute to the higher livelihood scores observed here, it is impossible to ascribe this to the governance model alone. However, qualitative and quantitative data from triangulation interviews lend weight to the idea that the co-management system in ACA, and particularly the coordinating role of the NTNC, has had a positive impact on livelihoods, especially through the creation of social capital, itself a form of influence. Despite outliers, exceptions, and a greater degree of devolution in SNP since 2002 (Silwal et al., 2022), this would seem to broadly confirm Ribot and Peluso’s (2003) theory of access, that access to influence does indeed shape access to assets.

Despite significant constitutional changes in Nepal since the data were collected, the relationship between PA governance and local livelihoods remains a critical issue for conservation in the country, the region, and the world. Given that several analyses have found that including rather than excluding local communities can lead to both effective conservation and socio-economic outcomes (Elleason et al., 2021; Oldekop et al., 2016), inclusionary approaches to PA governance should be prioritized wherever possible. Moreover, and particularly in the debate about protecting up to 50% of the world’s surface area for biodiversity (Dinerstein et al., 2017; Schleicher et al., 2019), socially-just conservation processes that simultaneously increase the feasibility and acceptability of protecting biodiversity are essential. More site-specific research critiquing PA governance via the mediums of assets and influence would help to navigate and reconcile these tensions and trade-offs between PA governance and livelihoods in the twenty-first century.

## Supplementary Information

Below is the link to the electronic supplementary material.

Supplementary Material 1 (DOCX 16.5 KB)

## Data Availability

No datasets were generated or analysed during the current study.
