# Supplementary material for: Access to Assets and Influence: Governance and Livelihoods in Protected Areas of the Annapurna and Everest Regions, Nepal
Source: Hum Ecol Interdiscip J. 2025 Sep 19;53(5):1069–85. doi: 10.1007/s10745-025-00643-4 (PMC12852147; doi:10.1007/s10745-025-00643-4)
Supplement: Supplementary file 1 — Supplementary Material 1 (DOCX 16.5 KB) [file 10745_2025_643_MOESM1_ESM.docx]

Supplementary information

*This supplementary information has not been peer reviewed.*

Title: Access to assets and influence: livelihoods and conservation governance in the Annapurna and Everest regions of Nepal

**Table S****1.** Trends in livestock and crop production for SNP and ACA based on triangulation interviews.

| **Aspects of livestock production** | | **SNP** | | **ACA** | |
| --- | --- | --- | --- | --- | --- |
|  |  | N = 26 | | N = 44 | |
|  |  | **N** | **%** | **N** | **%** |
| **Sale of surplus and/or commercial production** | | 19 | 73.1 | 20 | 45.5 |
| **Decreasing production** | | 7 | 10.0 | 11 | 25.0 |
| **Reasons for decrease** | No reason | 0 | 0.0 | 3 | 27.3 |
|  | Tourism | 7 | 100.0 | 3 | 27.3 |
|  | Migration | 0 | 0.0 | 2 | 18.2 |
|  | Motorised transport | 0 | 0.0 | 2 | 18.2 |
|  | Increased predation | 0 | 0.0 | 1 | 9.0 |
| **Aspects of crop production** | | | | | |
| **Sale of surplus and/or commercial production** | | 18 | 69.2 | 33 | 75.0 |
| **Decreasing production** | | 3 | 11.5 | 4 | 9.1 |
| **Reasons for decrease** | No reason | 1 | 33.3 | 1 | 25.0 |
|  | Tourism | 1 | 33.3 | 1 | 25.0 |
|  | Cheaper imports | 1 | 33.4 | 1 | 25.0 |
|  | Switch to cash crops | 0 | 0.0 | 1 | 25.0 |

**Linear model additional data S1.** Additional and diagnostic information for combined sample linear model explaining household Sustainable Livelihoods Index scores.

Potential explanatory variables not included in analysis due to small sample size in 'yes' category: income from wood (n=2); income from loans (n=10). Cronbach's Alpha not applicable for the Sustainable Livelihoods Index as its is not a scale measuring latent variables but an aggregation of a series of separate indicators. This model had the highest significant R² change score (.15, p = <.001) out of the nine models tested; variables excluded from final model due to lower R² change scores when included in successive models: other sources of income; NTFP income; study site.

**Diagnostics:**

1. **Correlation matrix** check for multicollinearity – no predictors highly correlated with each other (>.9).

2. **Model summary** assumption of independent errors met as Durbin-Watson statistic between 1 and 3 (1.291).

3. **Coefficients** check for multicollinearity - VIF values are less than 10 and not substantially greater than 1.

4. **Coefficients** check for multicollinearity – tolerance values are well above 0.2.

5. **Casewise diagnostics** check for bias – 5.26% cases >2, 1.48% cases >2.5, 3 cases >3.

6. **Cook's distance** check for outliers – no values above 1.

7. **P-P plot** check for normality – line curving round diagonal indicates non-normality, hence bootstrapping.

**Linear model additional data S2.** Additional and diagnostic information for linear model explaining household Sustainable Livelihoods Index scores in SNP.

Potential explanatory variables not included in analysis due to small sample size in 'yes' category: income from wood (n=1); income from loans (n=10); NTFP income (n=0); income from remittances (n=17). Potential predictor variable excluded from regression modelling due to equality of mean: livestock income (t = -0.15, *p* = .88). Cronbach's Alpha not applicable for the Sustainable Livelihoods Index as its is not a scale measuring latent variables but an aggregation of a series of separate indicators. This model had the highest significant R² change score (.18, p = <.001) out of the five models tested; variable excluded from final model due to a lower R² change score when included in the successive model: other income sources.

**Diagnostics:**

1. **Correlation matrix** check for multicollinearity – no predictors highly correlated with each other (>.9).

2. **Model summary** assumption of independent errors met as Durbin-Watson statistic between 1 and 3 (1.463).

3. **Coefficients** check for multicollinearity - VIF values are less than 10 and not substantially greater than 1.

4. **Coefficients** check for multicollinearity – tolerance values are well above 0.2.

5. **Casewise diagnostics** check for bias – 5.04% cases >2, 1.26% cases >2.5, one case >3.

6. **Cook's distance** check for outliers – no values above 1.

7. **P-P plot** check for normality – line curving round diagonal indicates non-normality, hence bootstrapping.

**Linear model additional data S3.** Additional and diagnostic information for linear model explaining household Sustainable Livelihoods Index scores in ACA.

Potential explanatory variables not included in analysis due to small sample size in 'yes' category: wood (n=1); loans (n=0); savings (n=22). Potential predictor variable excluded from regression modelling due to equality of mean: other sources of income (t = 0.76, *p* = .45). Cronbach's Alpha not applicable for the Sustainable Livelihoods Index as its is not a scale measuring latent variables but an aggregation of a series of separate indicators. This model had the highest significant R² change score (.24, p = <.001) out of the six models tested; variable excluded from final model due to lower R² change score when included in the successive model: NTFP income.

**Diagnostics:**

1. **Correlation matrix** check for multicollinearity – no predictors highly correlated with each other (>.9).

2. **Model summary** assumption of independent errors met as Durbin-Watson statistic between 1 and 3 (1.174).

3. **Coefficients** check for multicollinearity - VIF values are less than 10 and not substantially greater than 1.

4. **Coefficients** check for multicollinearity – tolerance values are well above 0.2.

5. **Casewise diagnostics** check for bias – 5.41% cases >2, 1.62% cases >2.5, two cases >3.

6. **Cook's distance** check for outliers – no values above 1.

**7. P-P plot check for normality –** line curving round diagonal indicates non-normality, hence bootstrapping.
